# Architecture of the AP2/clathrin coat on the membranes of clathrin-coated vesicles

**DOI:** 10.1126/sciadv.aba8381

**Published:** 2020-07-22

**Authors:** Oleksiy Kovtun, Veronica Kane Dickson, Bernard T. Kelly, David J. Owen, John A. G. Briggs

**Affiliations:** 1 MRC Laboratory of Molecular Biology, Cambridge Biomedical Campus, Cambridge CB2 0QH, UK.; 2 Structural and Computational Biology Unit, European Molecular Biology Laboratory, 69117 Heidelberg , Germany.; 3 Cambridge Institute for Medical Research, University of Cambridge, Hills Road, Cambridge CB2 0XY, UK.

## Abstract

Clathrin-mediated endocytosis (CME) is crucial for modulating the protein composition of a cell’s plasma membrane. Clathrin forms a cage-like, polyhedral outer scaffold around a vesicle, to which cargo-selecting clathrin adaptors are attached. Adaptor protein complex (AP2) is the key adaptor in CME. Crystallography has shown AP2 to adopt a range of conformations. Here, we used cryo–electron microscopy, tomography, and subtomogram averaging to determine structures, interactions, and arrangements of clathrin and AP2 at the key steps of coat assembly, from AP2 in solution to membrane-assembled clathrin-coated vesicles (CCVs). AP2 binds cargo and PtdIns(4,5)*P*_2_ (phosphatidylinositol 4,5-bisphosphate)–containing membranes via multiple interfaces, undergoing conformational rearrangement from its cytosolic state. The binding mode of AP2 β2 appendage into the clathrin lattice in CCVs and buds implies how the adaptor structurally modulates coat curvature and coat disassembly.

## Teaser

Cryo-electron tomography reveals the conformational changes and clathrin interactions of AP2 during coated vesicle assembly.

## INTRODUCTION

Clathrin-mediated endocytosis (CME) is a vital multistep process in all eukaryotes in which plasma membrane cargo proteins are first concentrated into a patch by clathrin adaptors, which are themselves clustered by interaction with a polymeric clathrin scaffold. The patch of membrane is deformed toward the cytoplasm and undergoes scission from the parent membrane to form a clathrin-coated vesicle (CCV), which subsequently delivers its cargo into the endocytic system [reviewed in (*1*, *2*)].

**Fig. 1:**
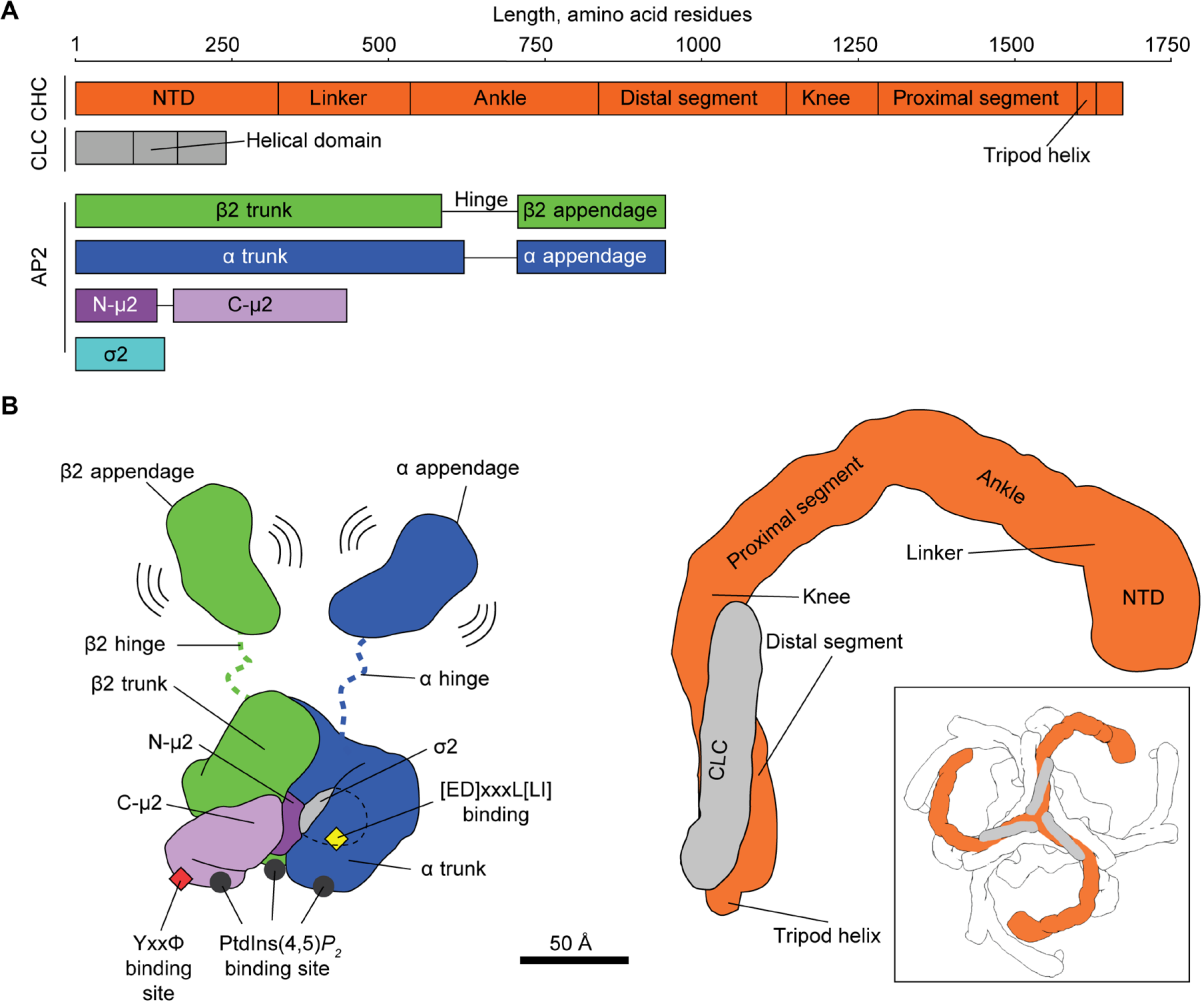
Overview of AP2 and clathrin structures. (**A**) Schematic representation of AP2 and clathrin polypeptide chains with marked domains. A length ruler (in amino acid residues) is given at the top. Note that the FLAP AP2 construct lacks α hinge and appendage regions, while the Core AP2 construct lacks α and β2 hinge and appendage regions. (**B**) Cartoon representation of the structures of AP2 (left) and clathrin (right). Subunits are color-coded as in (A) and known functional sites and domains are marked. The boxed panel shows a zoomed-out view of a clathrin triskelion with neighboring triskelia in white.

The clathrin lattice, containing mainly pentagonal and hexagonal openings, is formed from triskelia containing three copies of the clathrin heavy chain (CHC) and three copies of the clathrin light chain (CLC) (Fig. 1) [reviewed in (*1*)]. The CHC N-terminal domain (NTD) β-propeller contains multiple binding sites for clathrin box peptides, which are scattered throughout the unstructured regions of clathrin adaptors (*1*, *3*, *4*). The most abundant clathrin adaptor (*5*) is the 300-kDa heterotetrameric adaptor protein 2 (AP2) complex, consisting of α, β2, μ2, and σ2 subunits (Fig. 1). AP2 plays a central role in CME through binding plasma membrane phosphatidylinositol 4,5-bisphosphate [PtdIns(4,5)*P*_2_], the two most common cargo motifs (YxxΦ and [ED]xxxL[LI]), clathrin, and other regulatory/accessory proteins [reviewed in (*1*)].

AP2 is one of the first proteins to arrive at a forming CCV (*6*, *7*) and is also important in initiating clathrin polymerization (*6*, *8*, *9*). The favored although unproven model for AP2 and clathrin in CME suggests that cytosolic AP2 is closed and unable to bind cargo and that membrane-bound AP2 adopts a more open conformation in which cargo-binding sites are accessible (*10*, *11*). A closed conformation has been seen under nonphysiological conditions in crystals (*12*). A number of different possible structures for membrane-bound, cargo-binding “open” forms of AP2 complexes have been determined in the absence of membrane (*11*, *13*). Similarly, the highest-resolution structures of clathrin have been determined in the absence of a membrane and/or a characterized complement of folded adaptors. It therefore remains unclear how AP2 and clathrin interact with one another and the membrane to form CCVs. Here, we present the structures, and relative arrangements of AP2 and clathrin under physiological buffer conditions and in membrane-associated vesicle coats.

## RESULTS AND DISCUSSION

### AP2 structures

**Fig. 2:**
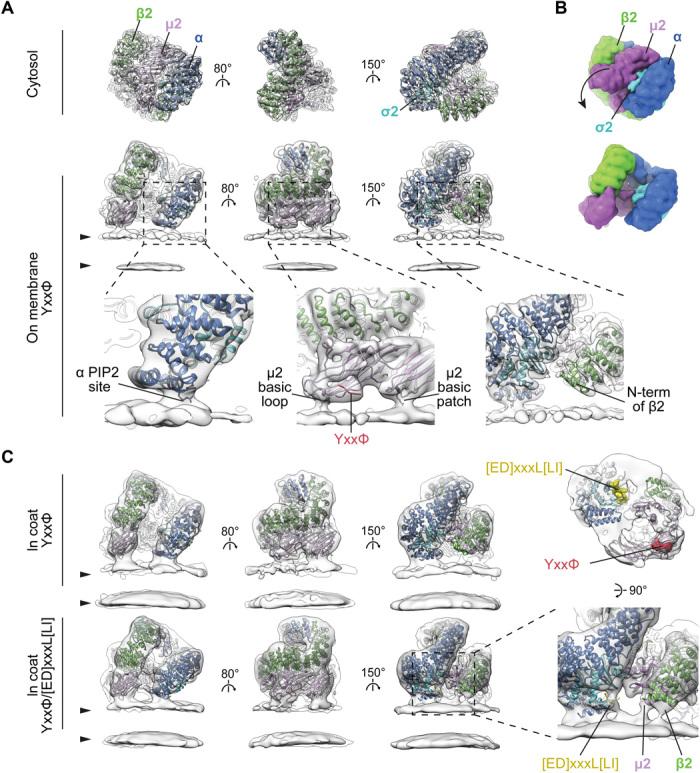
Conformational activation and membrane interactions of AP2. (**A**) Comparison of single-particle structure of the cytosolic closed form (top) with the tomography-resolved open form of AP2 on the membrane in the presence of YxxΦ cargo (bottom). EM maps (semitransparent gray) are fitted with corresponding ribbon models of AP2 color-coded by chain. Arrowheads indicate membrane leaflets. Close-ups show details of membrane contacts with α and μ2 subunits and the lack of contact with β2, with PtdIns(4,5)*P*_2_ sites and cargo peptides marked. The μ2 basic loop includes K167, Y168, R169, and R170, while the μ2 basic patch includes K350, K367, and R368. (**B**) Surfaces of the atomic models for cytosolic and membrane-recruited AP2 in (A) are shown to illustrate the rearrangement between cytosolic and membrane-bound forms. The arrow indicates the major movement of Cμ2 out of the bowl of AP2. (**C**) Conformation and membrane interactions of AP2 recruited to the membrane via YxxΦ or YxxΦ/[ED]xxxL[LI] cargo signals within assembled clathrin coats. Additional tilting of α in respect to the membrane is apparent when [ED]xxxL[LI] cargo is present (compare top and bottom panels in the left column). The close-up demonstrates new membrane contacts formed by β2 and μ2 subunits, and additional density in the cargo binding pocket when [ED]xxxL[LI] cargo is present. The panel above the close-up shows YxxΦ/[ED]xxxL[LI]-bound AP2 viewed from the membrane side with peptide-binding sites marked. See also fig. S2E for close-up views of the cargo-binding sites.

We assessed the conformation(s) adopted by recombinant AP2 cores in solution under physiological buffer conditions using single-particle cryo–electron microscopy (fig. S1A). The bulk of AP2 (~80%) adopted a closed conformation, the structure of which we determined to 3.8 Å. The remaining particles (~20%) did not refine to a reliable structure (i.e., no alternative open conformer was identified). The structure is similar to that seen under high-salt, high-pH conditions in the crystal structure containing inositol hexakis-phosphate (*12*): The C-terminal domain of μ2 (Cμ2) is located in the spatially complementary bowl created by the other subunits, while the YxxΦ and [ED]xxxL[LI] cargo motif-binding sites are blocked by portions of the β2 subunit (Fig. 2A and fig S2) (*11*).

Recombinant AP2 FLAP (full-length β2 subunit AP2 complex i.e. AP2 lacking the α appendage and the α hinge, which is proteolytically degraded in the *Escherichia coli* expression system; Fig. 1 and fig. S1E) is competent to mediate budding in vitro and avoids the presence of contaminating CCV components present in AP2 preparations from cells (*9*). We recruited AP2 FLAP (henceforth AP2) onto phospholipid membranes containing PtdIns(4,5)*P*_2_ and TGN38 YxxΦ cargo in the same physiological buffer conditions used for single-particle cryo–electron microscopy and imaged them by cryo–electron tomography. AP2 coated, and in some cases tubulated, the preexisting liposomes. We did not observe formation of buds or small vesicles. This suggests that AP2 alone does not drive membrane budding in this system (fig. S1B). We applied subtomogram averaging to determine the structure of AP2 to ~9 Å resolution (Fig. 2A and fig. S2). The structure closely resembles the previously determined crystal structure of the open conformation (*11*), although Cμ2 and the N-terminal portion of β2 are positioned approximately 5 Å further away from α (fig. S2C). Compared to the closed form, Cμ2 has moved to form a planar membrane-binding surface (Fig. 2B), and its movement with respect to the β2 N terminus has freed up the YxxΦ cargo binding pocket, which has a density consistent with that of a bound cargo peptide. The [ED]xxxL[LI] cargo motif-binding site on σ2 is accessible to membrane-embedded cargo owing to a movement of the N terminus of β2 relative to σ2. In the open form crystal structure (*11*), the [ED]xxxL[LI] site is occupied by the Myc-tag of a neighboring AP2. Our structure of AP2 on the membrane shows that the open conformation did not result from this or other crystal packing artifacts.

We observed three sites at which the AP2 electron density contacts the membrane: the proposed PtdIns(4,5)*P*_2_-binding site on the α subunit whose mutation abolishes AP2 membrane binding (*11*, *14*) and two putative PtdIns(4,5)*P*_2_-binding sites on Cμ2 (Fig. 2A), the simultaneous mutation of which inhibits membrane binding and reduces CCV nucleation (*11*, *15*). The first Cμ2 site corresponds to a basic loop (Fig. 2A) including K167, Y168, R169, and R170 following the μ2 linker helix, which undergoes an extended to helical transition between closed and open AP2 (*11*). This location suggests a direct physical connection between membrane binding and stabilization of the open AP2 conformation and points to interference with this linkage being the explanation for why an R170W mutation impairs CME and causes neurological defects (*16*). The second Cμ2 contact appears to be mediated by residues K350, K367, and R368 within a basic patch (Fig. 2A) also containing K330, K334, K352, K354, K356, and K365. We do not observe a membrane contact at the proposed β2 N-terminal PtdIns(4,5)*P*_2_-binding site (*11*, *15*).

We assembled both AP2 and clathrin onto YxxΦ cargo/PtdIns(4,5)*P*_2_-containing membranes. In contrast to the sample lacking clathrin, buds and vesicles were now formed with both AP2 and clathrin coating their membranes (fig. S1), indicating a role for clathrin scaffold recruitment in membrane curvature. The AP2 structure was identical up to the determined resolution (12 Å) to that seen in the absence of clathrin (Fig. 2C). Inclusion of an additional “dileucine” [ED]xxxL[LI]-containing cargo resulted in tilting of α, and movement of β2 away from σ2 to further expose its [ED]xxxL[LI]-binding site. Helices 2 and 5 in σ2 are poorly resolved, and the proposed β2 N-terminal PdIns(4,5)*P*_2_-binding site now contacts the membrane as does a region in the vicinity of helices 2 and 3 of μ2 (Fig. 2C).

When combined with published information, our data suggest a model whereby AP2 initially contacts the membrane in the closed conformation via PtdIns(4,5)*P*_2_-binding sites on α and β2. Liganding of the two Cμ2 PtdIns(4,5)*P*_2_-binding sites will shift the equilibrium to the open state in which the cargo-binding sites become unblocked. In vivo, this may be aided by the binding of FCHO1/FCHO2/sgip (*10*, *17*). AP2 can then “scan” the local membrane for cargo and its binding would then further stabilize an open form on the membrane. The binding of PtdIns(4,5)*P*_2_ and cargo are thus allosterically linked.

The transitions from cytosolic to single-cargo membrane-bound AP2, and from single- to double-cargo membrane-bound AP2, involve conformational flexing of the α and β2 solenoids. We did not observe previously described open+ (*13*), AP1-like hyper-open (*18*), or splayed, coat protein complex I (COPI)–like (*19*) forms (fig. S2D), suggesting that the open form is the lowest energy conformer for membrane-associated, nonphosphorylated AP2.

### AP2 distribution on membranes

We found the distribution of AP2 on the surface of membranes to be largely irregular, lacking long-range organization. On YxxΦ-only–containing membranes, ~20% of AP2s are dimerized (fig. S3A), their β2 N-terminal helices having apparently moved to participate in the interface. The dimerization interface is not similar to those proposed for AP1 multimerization via the small guanosine triphosphatase Arf1 (*20*). The AP2 distribution was unchanged by the addition of clathrin and, hence, by the formation of buds and vesicles. In vesicles and buds with both [ED]xxxL[LI] and YxxΦ cargoes, the dimeric form of AP2 is absent, likely because the N-terminal helix of β2 has moved to contact the membrane and can no longer contribute to dimerization interface. We did not observe a regular packing arrangement of AP2 in our assembled vesicles, excluding the possibility that clathrin organizes AP2 into a lattice, and also excluding AP2 oligomerization as an essential step in membrane curvature generation.

### Clathrin structure

**Fig. 3:**
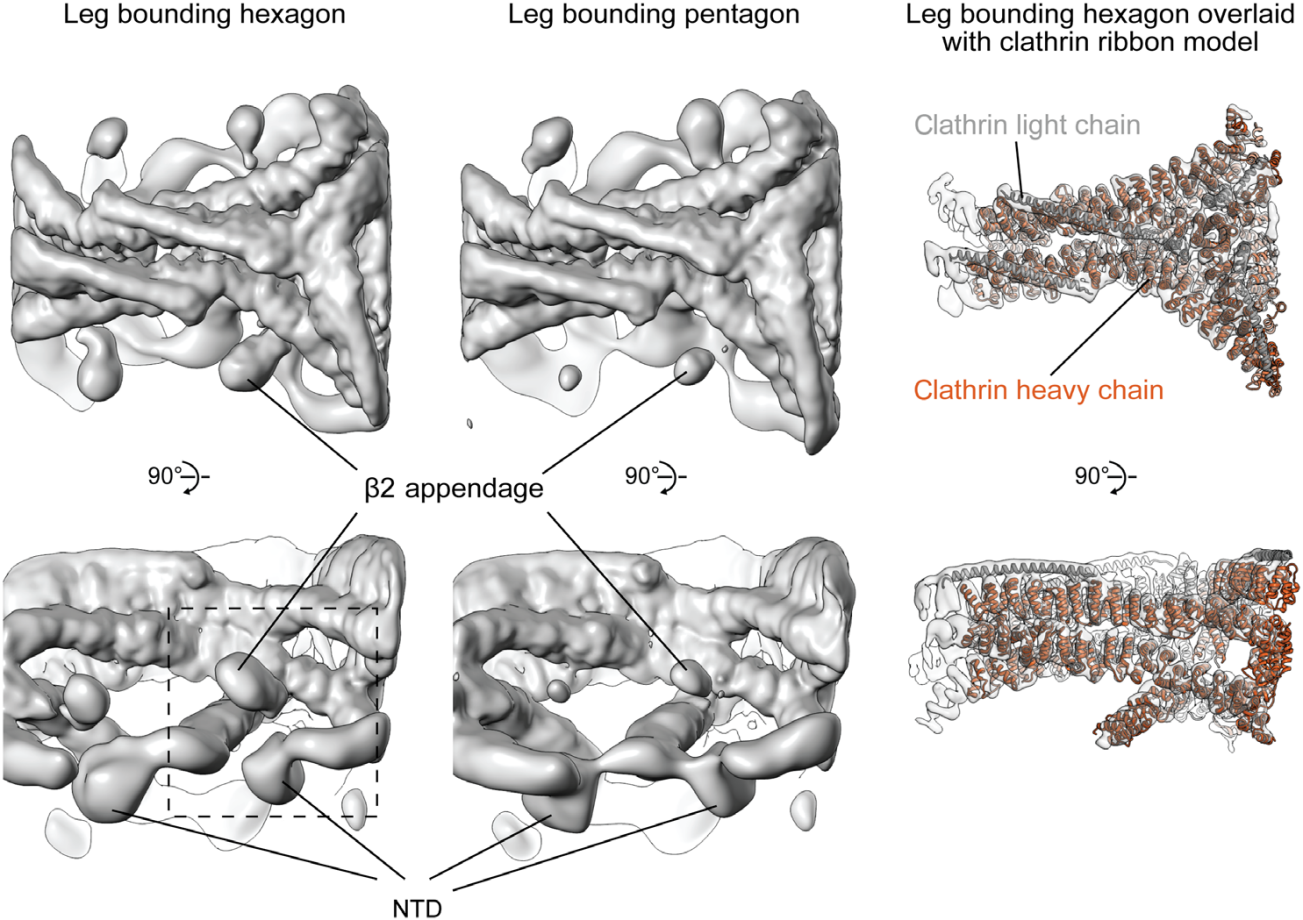
The structure of clathrin on coated membranes. EM maps (gray surface) of clathrin legs bounding hexagon and pentagon (see fig. S4 for details). The right column shows the EM map (sharpened to reveal high-resolution features) overlaid with a fitted ribbon model for the clathrin hub. N-terminal portions of CHC are clearly defined. Density corresponding to the AP2 β2 appendage indicates higher occupancy in hexagon than in pentagon maps (see also fig. S6). The dashed box indicates the region shown in Fig. 5.

**Fig. 4:**
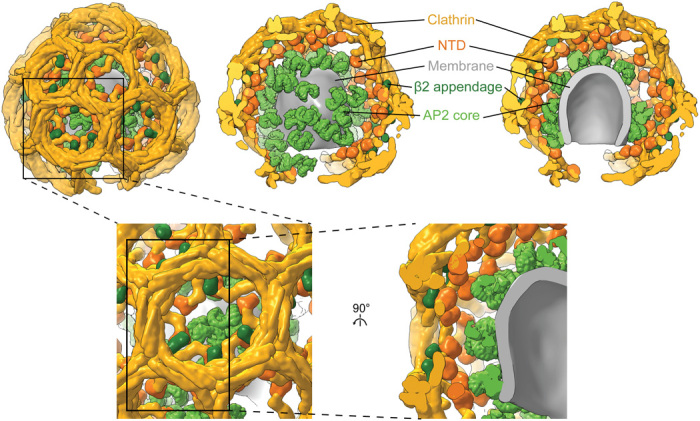
Experimental model of a coated bud formed on the YxxΦ cargo–containing membrane. The model of a representative bud was produced by placing densities for AP2 core (light green), AP2 β2 appendage (dark green), and clathrin legs (gold with NTDs colored orange) at positions and orientations determined by subtomogram averaging and classification (for β2 appendage) for that bud. AP2 core, β2 appendage, and clathrin densities were simulated on corresponding pseudoatomic models. The membrane was defined by segmenting the tomogram. (**Top**) The left panel shows the exterior of the bud. The front half of the clathrin cage is removed in the middle panel, revealing the random AP2 distribution on the membrane. The right panel shows a cross section through the entire bud. (**Bottom**) Close-up and cut-open views at the hexagon boxed in the top left panel.

**Fig. 5:**
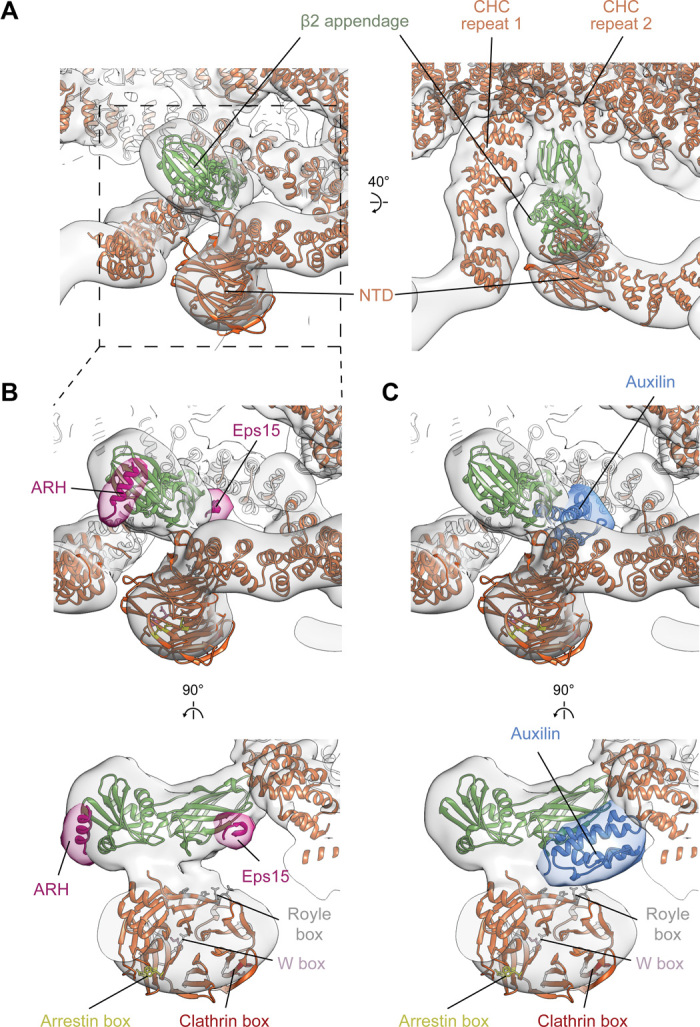
Interactions between clathrin and the AP2 β2 appendage domain. (**A**) Positions of NTD and the β2 appendage with respect to the clathrin lattice. Overlay of EM map of leg bounding hexagon enriched in β2 appendage (transparent gray; see fig. S6 for details) with fitted protein ribbon models. The β2 appendage makes contact with sites on three CHC: two in the ankle segment (CHC repeats 1 and 2) and one in the NTD. (**B**) Close-up of NTD and β2 appendage region indicating the binding sites of low-density lipoprotein receptor adaptor ARH and Eps15. ARH and Eps15 peptides are pink, positioned as in PDB 2g30 (*23*) and 2iv9 (*24*), together with a simulated 15-Å-resolution isosurface (transparent pink). Binding sites for clathrin boxes in the NTD are marked. (**C**) As in (B), showing the binding position of DNAJ auxilin domain in blue, as described in PDB 1xi5 (*25*).

We determined the structure of clathrin above AP2 in both the YxxΦ and YxxΦ/[ED]xxxL[LI] cargo–containing liposomes by subtomogram averaging: The structures were the same and thus were combined for further analysis (Materials and Methods). The clathrin legs were sorted into two classes according to whether the leg bounds a hexagonal or pentagonal cage face (fig. S4, A and B). Each class was averaged separately to generate structures at ~7.7 Å resolution (Fig. 3 and fig. S4, C and D). The CLC region that contacts CHC is well resolved, as are most of the leg and the helical hub of CHC. The resolution is lower toward the NTDs of CHC that extend downward toward the membrane, reflecting mobility of these domains, but is sufficient to position the full NTD. Our structures are generally consistent with those previously described from purified clathrin in solution in the absence of membranes (*21*, *22*), but by now resolving the NTDs (Figs. 3 and 4), we were able to derive the positions of peptide-binding sites within the clathrin cage (Fig. 5).

### Clathrin/AP2 relationship

In YxxΦ cargo-containing coated buds, the clathrin NTDs are positioned at a higher radius than AP2, and there are no preferential positions or orientations of AP2 relative to clathrin (Fig. 4 and fig. S5). Inclusion of ED]xxxL[LI] cargo or transition of the buds to vesicles causes clathrin to move toward the membrane, on average, by 10 to 15 Å, after which most NTDs are at a lower radius than the top of AP2 (fig. S5). In these cases, AP2 is preferentially located in the gaps between the NTDs, though with no preferred rotational orientation. This suggests that the preferred localization is due to steric clashing rather than specific interaction. It is not clear to us what causes the movement of clathrin toward the membrane.

### The β2 appendage

Between the clathrin NTDs and the undersides of the triskelia, we observed a “bean-shaped” density that is not part of clathrin (Fig. 3). We applied principal components analysis (PCA) to sort the datasets according to the presence or absence of this additional density (fig. S6A) and averaged positions where it was present within a hexagon, obtaining a structure corresponding to the AP2 β2 appendage (Materials and Methods) (Fig. 5A and fig. S6). The β2 hinge is not visible—most of it will be flexible, while short regions bound to a subset of clathrin molecules would be too small to be amenable to image-based sorting. When comparing the density and occupancy of the β2 appendage between sites oriented toward the centers of either hexagons or pentagons, we found preferential binding in hexagonal faces (more than double the apparent occupancy at pentagonal faces; fig. S6A).

β2 appendages link CHC repeat 1 in one clathrin molecule, CHC repeat 2 in a second clathrin molecule (both in the ankle region), and the NTD of a third clathrin molecule (Fig. 5A). This positions the β2 appendage such that its C-terminal platform subdomain is freely accessible to the Fxx[FL]xxxR helical motifs on the unstructured portions of secondary adaptors such as autosomal recessive hypercholesterolemia (ARH) protein, arrestins, and epsins (Fig. 5B) (*23*) rather than being blocked as speculated (*24*). Another motif-binding site, recognized by regulatory/accessory proteins including Eps15, is located on the β2 appendage sandwich domain close to the point of interaction with CHC repeat 2 (Fig. 5B).

The β2 appendage is dispensable for clathrin polymerization in vitro (fig. S7A). Binding of the β2 appendage to the clathrin scaffold therefore likely reflects roles in regulating membrane curvature, assembly, and/or disassembly in vivo. The β2 appendage clathrin-binding site we can now see is adjacent to the binding site of the DnaJ domain of auxilin proposed on the basis of single-particle electron microscopy studies (Fig. 5C) (*25*, *26*). Auxilin can induce coat disassembly by recruiting HSC70 (*27*) [and reviewed in (*28*)], which also could indirectly modulate levels of cargo incorporation by AP2 (*29*). We now see that auxilin would occupy a cavity bounded by the β2 appendage, an NTD, and clathrin ankles. On binding at the proposed position, an auxilin DNAJ domain would overlap with the binding site for Eps15 on the β2 appendage and would be able to interact with the NTD [consistent with (*24*)] by contacting the “Royle” box binding groove (Fig. 5, B and C) (*4*). Interactions between clathrin, auxilin, the β2 appendage, and Eps15 at this regulatory nexus could be competitive or synergistic, coupling the concentration of adaptors (and therefore cargo) to dynamic rearrangement of the clathrin lattice. β2 appendage preferentially binds to hexagons, while bending of the clathrin lattice requires increasing the fraction of pentagons: Hence, expulsion of β2 appendage from its location in the coat and increasing lattice curvature will favor each other. Our in vitro system functionally reconstitutes AP2/clathrin-dependent vesicle formation, but it should be noted that the α appendage, which does not bind clathrin directly, is not present, nor are other regulatory components such as clathrin assembly lymphoid myeloid leukaemia (CALM) protein or epsin. In vivo, these and other components could also contribute to modulation or regulation of coat assembly and curvature. Together, our observations suggest that cargo binding, coat curvature, and dynamic assembly and disassembly of the coat could be connected and controlled by reconfiguring low-affinity, high-avidity interactions between clathrin, adaptors, and auxilin at a single site on the clathrin scaffold.

## MATERIALS AND METHODS

### Preparation of reagents

To produce recombinant AP2 proteins, bacterial expression and purification, clathrin purification from porcine brain, liposome pelleting, lipopeptide coupling, and liposome preparation were carried out as in (*9*, *11*). AP2 FLAP lacks the α subunit hinge and appendage (Fig. 1 and fig. S1E). Δβ2 appendage FLAP additionally lacks the β2 appendage but is extended beyond the hinge region with an inert, unstructured region comprising 106 residues taken from the Enterobacteria phage f1 attachment protein G3P (residues 220 to 326, UniProtKB P69169), followed by a decahistidine tag. This extension facilitates purification of a fully intact β2 hinge region, which is otherwise cleaved.

For clathrin pulldown experiments, glutathione *S*-transferase (GST)–tagged adaptors (for capture on glutathione Sepharose beads) were constructed in pGEX-4T2 by genetically fusing fragments of β2 to GST. GST-β2hingeapp comprises the hinge and appendage regions of β2 fused to GST, as described previously (*9*). GST-β2hinge comprises the extended β2 hinge as described above for the “Δβ2 appendage FLAP” construct, fused to GST. As a non–clathrin-binding control, the green fluorescent protein was fused to GST. Fusion proteins were expressed and purified by standard techniques, using overnight expression at 22°C in BL21(DE3)pLysS cells.

All lipids were purchased from Avanti Polar Lipids. The YxxΦ cargo signal was derived from TGN38 protein with sequence CKVTRRPKASDYQRL, and the [ED]xxxL[LI] signal was derived from phosphorylated CD4 with sequence CHRRRQAERM(SPhos)QIKRLLSEK. N-terminal cysteines were used for lipid coupling to maleimide lipids.

### Glutathione Sepharose pulldown assays

Clathrin (0.8 μM) and adaptor (1.5 μM) were mixed in HKM buffer [25 mM Hepes (pH 7.2), 125 mM potassium acetate, and 5 mM magnesium acetate], total volume 100 μl, and incubated at 21°C for 30 min. Fifty-microliter aliquots of glutathione Sepharose beads (GE Healthcare) were washed four times in HKM/T buffer (HKM buffer supplemented with 0.05% Tween-20) before resuspension in the reaction mixtures. After a further 30 min of incubation at 21°C, with continuous gentle inversion to prevent settling, the beads were pelleted by centrifugation, supernatants were removed and retained for gel analysis, and the beads were washed four times in HKM/T before resuspension in 100 μl of HKM buffer. The pellet (“p”) and supernatant (“s”) samples were supplemented with 33 μl of 4× SDS gel loading buffer, heated to 37°C for 30 min with occasional vortexing, and finally heated to 95°C for 5 min before analysis by SDS-PAGE. Results are shown in fig. S7B and are representative of three experimental replicates.

### Single particle cryo–electron microscopy and model building

Purified AP2 core was reconstituted into physiological buffer HKM2 [10 mM Hepes KOH (pH 7.2), 120 mM KOAc, and 2 mM MgCl_2_] with 0.5 mM dithiothreitol and applied to Quantifoil grids (R1.2/1.3, 300 Cu mesh). Grids were glow-discharged for 60 s at 25 mA using a Pelco EasiGlow before application of 3.5 μl of sample (0.4 mg/ml) and plunge-freezing using a Vitrobot Mark IV (FEI Company) operated at 4°C and 95% humidity. Data collection was carried out on a Titan Krios transmission electron microscope (FEI/Thermo Fisher Scientific) operated at 300 keV, equipped with a Falcon 3EC direct detector (FEI/Thermo Scientific) in counting mode. Automated data acquisition was performed using FEI/Thermo Fisher Scientific EPU software at a nominal magnification of ×75,000, which corresponds to a pixel size of 1.065 Å per pixel. Dose-fractionated movies were acquired using 60-s exposures and 60 fractions at a dose of 0.54 e^−^ Å^−2^ s^−1^ with a defocus range of −1.9 to −3.1 μm (summarized in table S1).

Data quality assessment, movie frame alignment, estimation of contrast-transfer function parameters, particle picking, and extraction were carried out using Warp (*30*). Particle images (120,000) were extracted with a box size of 240 pixels and imported into CryoSPARC (*31*) for two-dimensional (2D) classification and ab initio model building. 3D refinement, filtering, and sharpening (*B* factor, −200) was carried out using the Homogeneous Refinement utility in CryoSPARC, including Fourier shell correlation (FSC) mask auto-tightening, followed by local resolution calculation by gold-standard FSC threshold (0.143 threshold) using the refined mask and an adaptive window factor of 25 (fig. S2, A and B).

The previously determined “closed” conformation of the AP2 core complex [Protein Data Bank (PDB) ID 2vgl] was fitted into the final cryo–electron microscopy volume using UCSF Chimera (*32*) and the phenix.dock_in_map function of the Phenix Software suite (cross-correlation = 0.83) (*33*). The structure was refined using phenix.real_space_refine and found to have an overall RMSD = 1.147 versus the 2vgl coordinates with no major structural rearrangement (fig. S2C and table S1).

### Cryo–electron tomography sample preparation and data acquisition

To reconstitute AP2 membrane recruitment and clathrin/AP2 membrane budding, purified AP2 FLAP and clathrin were incubated with synthetic liposomes. The liposomes contained 10% brain PtdIns(4,5)*P*_2_ and 10% 1,2-dioleoyl-sn-glycero-3-phospho-l-serine in a 1-palmitoyl-2-oleoyl-sn-glycero-3-phosphocholine/1-palmitoyl-2-oleoyl-sn-glycero-3-phosphoethanolamine (3:2) mixture, with 3% content of each cargo lipopeptide. In vitro budding reactions contained 1.4 μM AP2, 0.7 μM clathrin, and 0.5 mg/ml of 400 nm extruded liposomes in HKM2 buffer with 5 mM β-mercaptoethanol. AP2 and liposomes were incubated for 5 min at room temperature, clathrin was then added, and the reaction was incubated for a further 15 min at room temperature followed by 10-min incubation at 37°C. For the reaction without clathrin, liposomes were prepared by extrusion through a 200-nm filter, and the clathrin addition was omitted. Ten-nanometer gold fiducial markers in HKM2 buffer were added to the reaction (1:10 fiducials to the reaction volume ratio), and 3 μl of this mixture was back side blotted for 3 s at relative humidity 98% and temperature 18°C on a glow-discharged holey carbon grid (CF-2/1-3C, Protochips), before plunge-freezing in liquid ethane (Leica EM GP2 automatic plunger). Dose-symmetrical tilt series acquisition (*34*) was performed on an FEI Titan Krios electron microscope operated at 300 kV with a Gatan Quantum energy filter with a slit width of 20 eV and a K2 direct detector operated in counting mode. The total exposure of ~130 e^−^/Å^2^ was equally distributed between 41 tilts. Ten frame movies were acquired for each tilt. The details of data collection are given in table S2.

### Raw image processing and tomogram reconstruction

The raw movies were corrected for detector gain and pixel defects, and aligned and integrated using alignframes from the IMOD package (*35*). A few tilt series with tracking errors or large beam-induced sample movements were discarded. In addition, a small number of defective high-tilt images (identified by blur, tracking error, large objects like grid bar, or contaminations coming in the field of view) were discarded. Tilt series were low-pass–filtered according to the cumulative radiation dose (*36*) and aligned using fiducial markers in the IMOD package. Non–contrast transfer function (CTF)–corrected, four times binned tomograms were reconstructed by weighted back-projection in IMOD. For 3D CTF-corrected tomograms, per-tilt defocus estimation was performed in CTFPLOTTER on non–dose-filtered tilt series, and correction and reconstruction were done using novaCTF (*37*) with 15-nm strip width. Tomograms were binned by two, four, and eight times (hereafter called bin2, bin4, and bin8 tomograms) with anti-aliasing.

### Subtomogram alignment

Subtomogram alignment and averaging were performed using MATLAB (MathWorks) functions adapted from the TOM (*38*), AV3 (*39*), and Dynamo packages (*40*) essentially as described previously, using a modified wedge mask representing the amplitudes of the determined CTF and applied exposure filters at each tilt (*41*, *42*). Table S3 summarizes data processing parameters.

#### Tracing decorated membrane and initial subtomogram positions

To define initial positions of subtomograms, centers, and radii of coated vesicles, buds and tubules were manually marked in bin4 tomograms using a Chimera plug-in (*43*). No selection was made according to size. These measurements were used to define geometrical shapes: flexible tubes (for coated tubules) and spheres (for coated buds and vesicles). Subtomogram positions were then defined on the surface of these shapes (for AP2) or 120 Å above it (for clathrin) with uniform sampling at every ~45 or ~110 Å for AP2 and clathrin, respectively. Initial subtomogram orientations were calculated to be normal to the shapes’ surfaces with random in-plane rotation.

#### Ab initio reference generation

Subtomograms were extracted at the initial positions from bin8 tomograms and averaged according to their initial orientations. Subtomograms were then aligned to this average in the direction perpendicular to the membrane and averaged to generate a reference in which density layers corresponding to the lipid bilayer, adaptor, and clathrin layers were visible.

A subset of the data (three tomograms) was then aligned against this reference. For AP2, a Gaussian-blurred sphere 80 Å in diameter was first added to the adaptor layer to assist the convergence of alignment and a cylindrical mask was applied passing the AP2 layer and the membrane. Three iterations of alignment were performed with a 10° angular search increment and a 35-Å low-pass filter. For clathrin, a cylindrical mask was applied passing the clathrin layer and excluding the membrane layer. Four iterations of alignment were performed with a 5° angular search increment and a 47-Å low-pass filter. The resulting average was shifted, rotated, and threefold symmetrized to place the clathrin triskelia in the center of the box. In this manner, separate starting references were generated for AP2 and clathrin.

#### Subtomogram alignment

These references were then used to align the complete datasets with the same alignment parameters. Upon alignment convergence, oversampling was removed by selecting a single subtomogram with the highest cross-correlation score within a distance threshold of 71 or 100 Å for AP2 and clathrin, respectively.

Subtomograms were then sorted into identically sized odd and even subsets of coated structures. Subtomograms were extracted from bin4 tomograms and subsequent alignment was performed independently on the odd and even subsets. The search space and increments for angular and spatial parameters were gradually decreased, and the low-pass filter was gradually moved toward higher resolution. Upon convergence of subtomograms from bin4 tomograms, alignment was continued using bin2 and finally bin1 tomograms. Visibly misaligned subtomograms were removed using a cross-correlation cutoff threshold manually selected for each tomogram. Resolution was monitored by FSC. The final maps were sharpened with empirically determined *B* factor with local low-pass filtering using relion_postprocess from the Relion 3.0 package (*44*).

For AP2 alone, after convergence, focused alignment was performed by local masking of either α, β2/μ2, or the C termini of α and β2. The focused maps were multiplied by their alignment mask, summed, and divided by the sum of all three local masks to obtain the final EM map. Focused alignment was not performed for AP2 in clathrin coats due to insufficient signal. Final maps were multiplied by a soft cylindrical mask to remove diffuse density from neighboring AP2 molecules.

For clathrin, before alignment in bin2 tomograms, we performed symmetry expansion using dynamo_table_subbox from the Dynamo package (*40*) to generate subtomograms centered at individual clathrin legs and further alignment iterations were performed on individual legs.

#### Classification of subtomograms

We applied PCA to sort subtomograms by β2 appendage occupancy. The PCA was performed on wedge-masked difference maps (*45*) with calculations implemented in MATLAB using code adapted from PEET and Dynamo packages (*40*, *45*). The first eigencomponent of this PCA correlated with the β2 appendage density in the subtomograms and could be used to sort the data according to β2 appendage occupancy. We sorted subtomograms bounding pentagons or hexagons of the clathrin lattice based on the positions of their neighbors (fig. S4A).

To produce a β2 appendage–enriched subtomogram class, hexagon- and pentagon-bounding subtomograms were split into 10 equally sized classes according to β2 appendage occupancy (fig. S6A). The four classes of hexagon-facing legs with highest β2 appendage occupancy were combined together and the combined class was used for further alignment to derive the EM map resolving the β2 appendage density in the clathrin cage.

All EM maps generated from the two clathrin datasets (with YxxΦ and YxxΦ/[ED]xxxL[LI] cargoes) were essentially identical. Both clathrin datasets were therefore combined for further alignment in bin2 and bin1.

### Spatial distributions of AP2 and clathrin

Plots showing the distribution of proteins relative to their neighbors were created as in (*46*). Plots of the distribution of AP2 relative to AP2 (fig. S3A) relate the centers of AP2 complexes. These plots were used to identify AP2 dimers in the dataset of AP2 on the YxxΦ cargo–containing membrane, which appear as a peak in the distribution containing ~20% of total AP2 (marked by an arrow in fig. S3A). To derive a structure of the dimer, subtomograms containing dimers were aligned in bin4 without symmetrization and then in bin2 with twofold symmetrization until convergence.

Plots of the distribution of clathrin relative to clathrin (fig. S4A) relate the CHCR3 regions of distal clathrin segments. These plots were used to separate clathrin legs that bound hexagons and pentagons in the clathrin cage (fig. S4).

Plots of the distribution of AP2 relative to clathrin relate the center of AP2 and the tripod region of the clathrin leg. Plots of the distribution of clathrin relative to AP2 relate the center of AP2 and the membrane-facing edge of clathrin NTDs. These plots were used to analyze spatial relationships between the adaptor and clathrin components of the coat (fig. S5).

Linear profiles of the radial distribution of AP2 relative to the clathrin tripod were derived by integrating the AP2 distribution plot along the axis of a cylindrical mask with a radius of 54 Å. The mean and standard deviation of the linear profiles were calculated by Gaussian fitting (Python, Matplotlib).

### Model building in EM maps determined by subtomogram averaging

Rigid-body docking was performed using the Chimera package (*32*). Flexible fitting was performed using molecular dynamics flexible fitting (MDFF) in the NAMD package (*47*) and Phenix (*33*), constraining secondary structure elements. For AP2, flexible fitting of the crystal structure of the open form (PDB ID 2xa7) (*11*) was performed using NAMD followed by geometry refinement in Phenix. For CLC and the C-terminal region of CHC, fitting of PDB ID 6sct (residues 649 to 1596) (*22*), together with PDB ID 1xi4 (residues 545 to 659) (*21*), was performed into the map of leg bounding a hexagon (fig. S4C) using Phenix. For the NTD ankle region of CHC (residues 1 to 541), fitting was performed using (PDB ID 1xi4) (*21*) into a map of leg bounding a hexagon enriched in the β2 appendage (fig. S6C) using NAMD. This was done because the NTD ankle regions are better resolved in the β2 appendage–enriched map compared to the map representing all hexagon bounding legs, but are nevertheless at low resolution. PDB ID 1xi4 is a C-alpha trace model based on Electron Microscopy Data Bank (EMDB) entry EMD-5119, and to allow MDFF, side chains were added to the model using PD2 ca2main online server (*48*). NTDs were constrained as a single domain. The geometry of the composite model of the NTD ankle was refined using Phenix. The β2 appendage [PDB ID 1e42, (*49*)] was fit as a rigid body using Chimera. To display the position of the DNAJ domain of auxilin, the model of NTD/DNAJ taken from PDB ID 1xi5 (*25*) was overlaid with the clathrin NTD from our pseudoatomic model.

## Supplementary Material

aba8381_SM.pdf

## SUPPLEMENTARY MATERIALS

Supplementary material for this article is available at http://advances.sciencemag.org/cgi/content/full/6/30/eaba8381/DC1

## Appendix

View/request a protocol for this paper from *Bio-protocol*.
