# Supplementary material for: Architecture of the AP2/clathrin coat on the membranes of clathrin-coated vesicles
Source: Sci Adv. 2020 Jul 22;6(30):eaba8381. doi: 10.1126/sciadv.aba8381 (PMC7375805; doi:10.1126/sciadv.aba8381)
Supplement: aba8381_SM.pdf [file aba8381_SM.pdf]

Science Advances
AAAS

advances.sciencemag.org/cgi/content/full/6/30/eaba8381/DC1

Supplementary Materials for

Architecture of the AP2/clathrin coat on the membranes of clathrin-coated vesicles

Oleksiy Kovtun, Veronica Kane Dickson, Bernard T. Kelly*, David J. Owen*, John A. G. Briggs*

*Corresponding author. Email: btk1000@cam.ac.uk (B.T.K.); djo30@cam.ac.uk (D.J.O.); jbriggs@mrc-lmb.cam.ac.uk (J.A.G.B.)

Published 22 July 2020, *Sci. Adv.* **6**, eaba8381 (2020)
DOI: 10.1126/sciadv.aba8381

This PDF file includes:

Figs. S1 to S7
Tables S1 to S3

Supplementary Materials

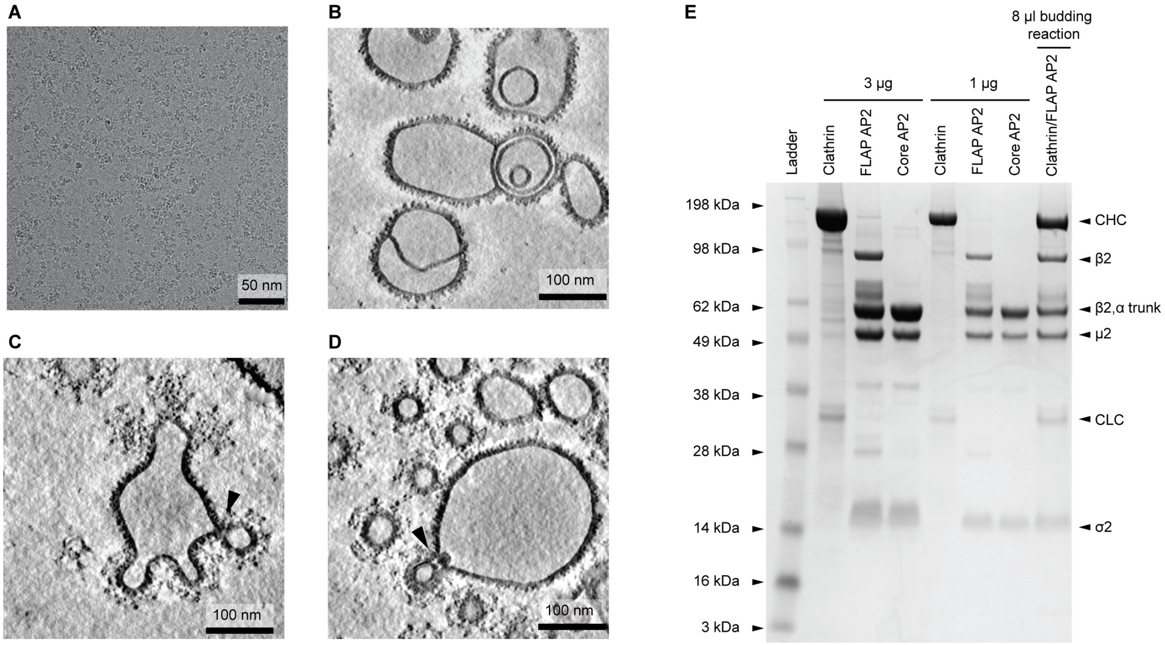

Fig. S1. Representative EM images of datasets used for structural characterisation and PAGE characterisation of protein preparations and budding reactions. A. A cryo-EM image of AP2 in buffer mimicking physiological conditions (see methods). **B.** Slices through a tomogram of AP2 recruited to membranes containing YxxΦ cargo. **C.** Clathrin/AP2 coats assembled on membranes containing YxxΦ cargo. **D.** Clathrin/AP2 coats assembled on membranes containing YxxΦ and [ED]xxxL[LI] cargo. In **B-D**, AP2 appears as a dense speckled coat on the membrane. Clathrin forms polygonal cages around the highly curved membranes of buds and vesicles. Coated buds often have narrow necks (arrowheads). Small vesicles only appear upon the addition of clathrin, suggesting that clathrin-coated vesicles formed via scission of their narrow necks. **E.** Electrophoretic separation in 4-12% NuPAGE gel in MOPS buffer (Invitrogen) of purified clathrin, AP2 and the complete budding reaction along with a molecular size ladder. FLAP AP2 lacks α hinge and appendage regions. Core AP2 lacks α and β2 hinge and appendage regions.

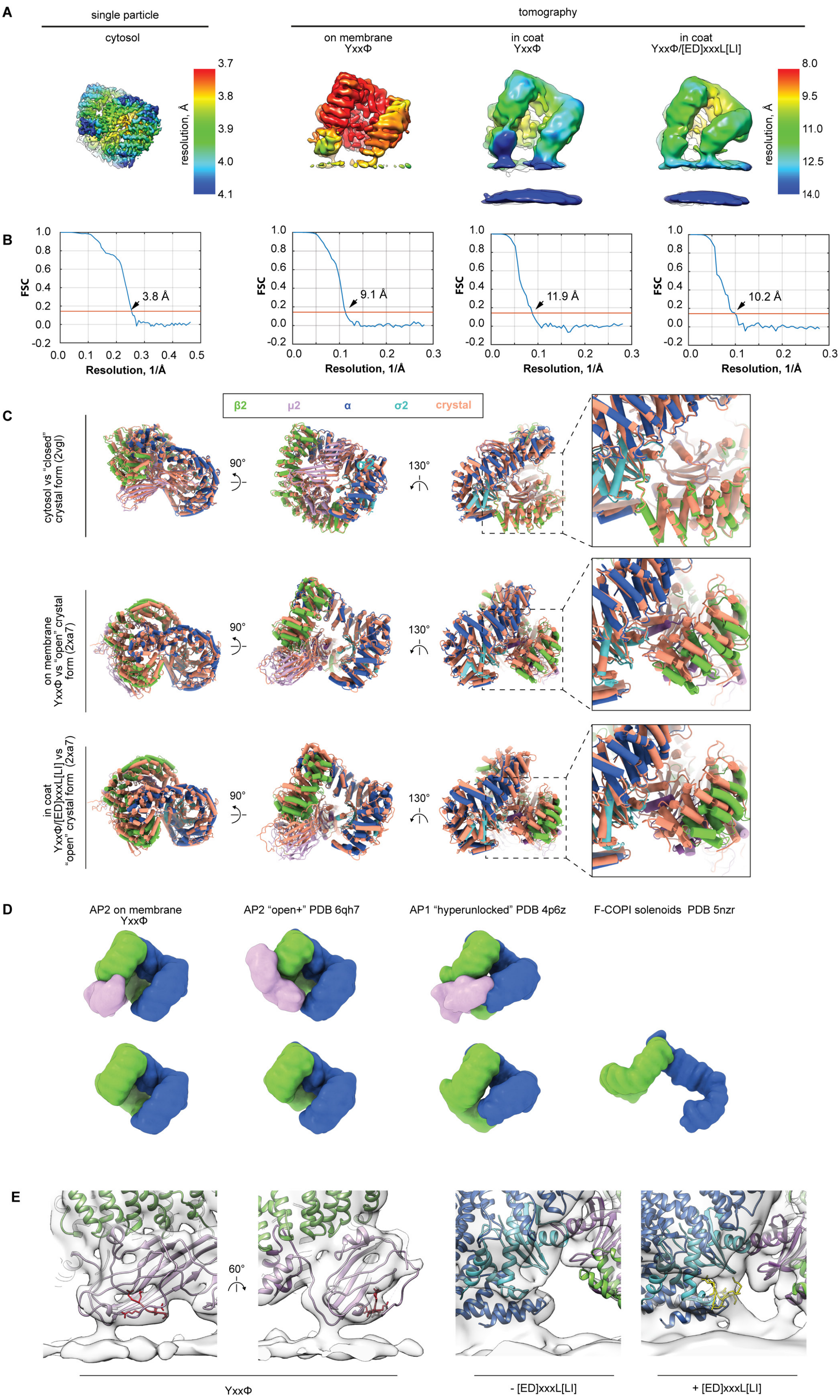
A
single particle
cytosol
tomography
on membrane YxxΦ
in coat YxxΦ
in coat YxxΦ/[ED]xxxL[LI]
resolution, Å
3.7
3.8
3.9
4.0
4.1
8.0
9.5
11.0
12.5
14.0
B
FSC
Resolution, 1/Å
3.8 Å
9.1 Å
11.9 Å
10.2 Å
C
β2
μ2
α
σ2
crystal
cytosol vs "closed" crystal form (2vgl)
on membrane YxxΦ vs "open" crystal form (2xa7)
in coat YxxΦ/[ED]xxxL[LI] vs "open" crystal form (2xa7)
90°
130°
D
AP2 on membrane YxxΦ
AP2 "open+" PDB 6qh7
AP1 "hyperunlocked" PDB 4p6z
F-COPI solenoids PDB 5nzr
E
60°
YxxΦ
- [ED]xxxL[LI]
+ [ED]xxxL[LI]

Fig. S2. Further characterization of the structure of AP2 on the membrane. A. EM maps colored by the local resolution for the four AP2 forms determined in this work. **B.** The corresponding FSC plots with arrows indicating the resolution at 0.143 FSC criterion. **C**. Overlays of AP2 structures modelled based on the EM maps in **A** and related crystal structures of the closed (2vgl, [4]) and open (2xa7, [5]) forms. Panels in the right column show close up views of the N-terminal region of β2. The β2 position is essentially the same in the cytosolic and closed crystal form of AP2, but appears more open in membrane-bound AP2 than in the "open" crystal form. **D.** Comparison of the structure of YxxΦ-cargo bound AP2 on a membrane with the "open+" conformation of AP2 (6qh7, [6]), the hyper-unlocked conformation of AP1 (4p6z, [7]), and the membrane-bound F-COPI α-solenoids (5nzr, [8]). The top row shows surface models of the alpha-solenoids of the large subunits and the Cμ2 domain, while the bottom row omits the Cμ2 domain (the Cμ2-homologous domain from F-COPI is not rigidly positioned relative to the solenoids). Subunits are colored as in panel **C**. **E.** Close up views of the cargo binding pockets, showing the occupied YxxΦ cargo pocket (from **Fig 2A**) in AP2 on YxxΦ cargo-containing membrane ("YxxΦ") and the [ED]xxxL[LI] cargo pocket in the clathrin/AP2 coat either unoccupied on membranes containing only YxxΦ cargo (**"-**[ED]xxxL[LI]") or occupied on membranes containing both YxxΦ and [ED]xxxL[LI] cargoes (**"**+[ED]xxxL[LI]", from **Fig. 2C)**. Cargo peptides represented as stick models. Coloring as in **Fig. 2**.

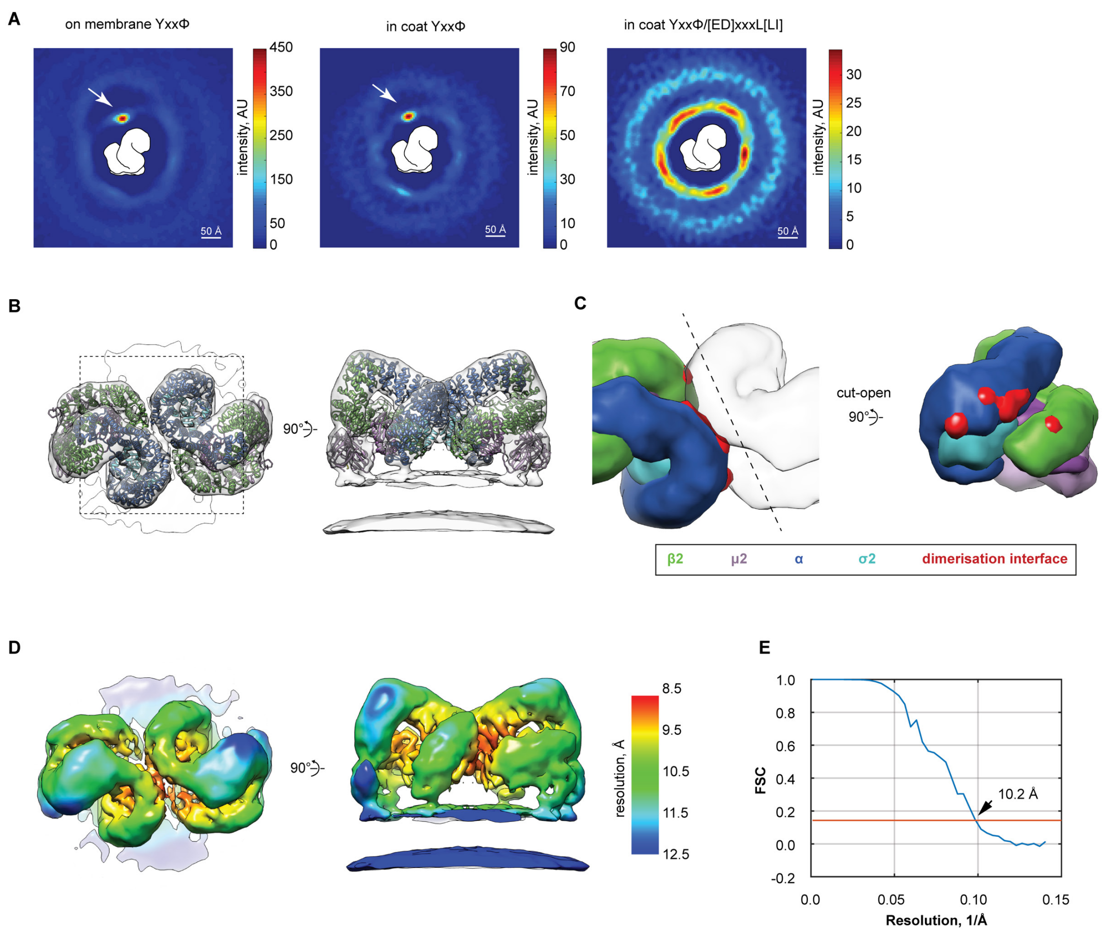

Fig. S3. Distribution of neighboring AP2s on membranes. A. Plots ("neighbor plots") show the spatial distribution of the centers of neighbouring AP2 molecules for the entire dataset of a specific type. High intensity indicates that a neighbouring AP2 molecule is found with high frequency at that position in space relative to the central AP2 molecule (outlined in white). The neighbor plot is three dimensional - for visualisation, a 60 Å thick central slab of the neighbor plots has been integrated perpendicular to the membrane and coloured by intensity. Arrows indicate preferred positions of neighbors corresponding to an AP2 dimer. The dimer is frequent on membranes containing YxxΦ-cargo (**left** and **center panels**) but not membranes containing YxxΦ/[ED]xxxL[LI] cargo (**right panel**). **B**. Averaging all AP2 molecules which have a neighbor at the dimer position generates a structure of the dimer. The EM structure of the dimer (transparent isosurface) has been rigid-body-fitted with two copies of the structural model for AP2 on YxxΦ-cargo membranes. The dashed box indicates the region at the interface in shown in **C. C.** Surface model of the AP2 dimer with one monomer made transparent for clarity. Dashed line indicates the position of the frontal viewing plane in the cut-open view (right). Contacting areas between the neighboring AP2 molecules in the dimer (defined as within a 6 Å distance threshold) are colored in red. **D.** The EM map of the AP2 dimer colored by local resolution. **E.** the corresponding FSC plot.

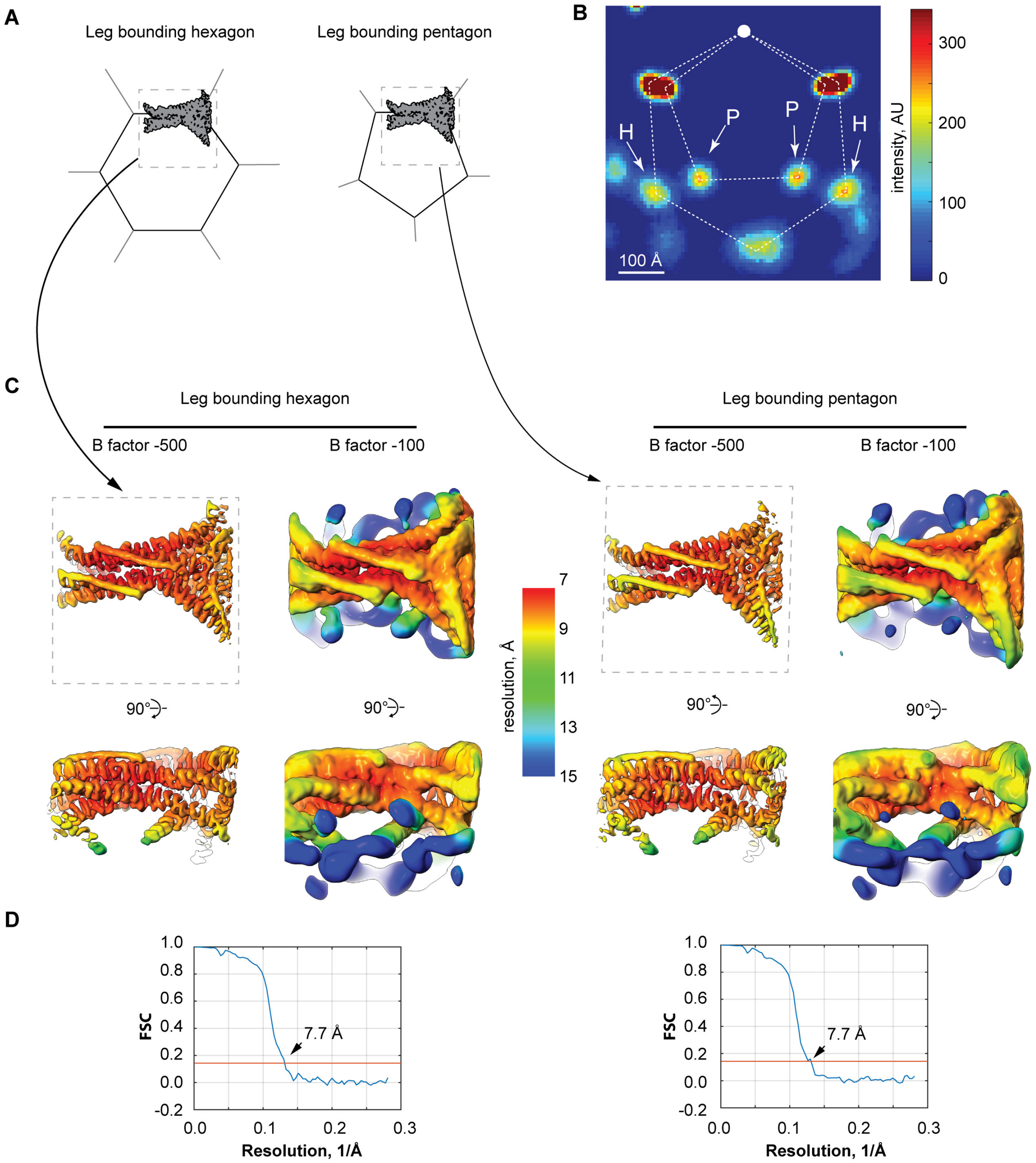

Fig. S4. EM maps of clathrin legs bounding hexagons and pentagons. A. A schematic representation of subtomograms containing a clathrin leg bounding a hexagon (**left**) or a pentagon (**right**) of the clathrin cage. The dotted boxes indicate boundaries of subtomograms with a cartoon representation of the clathrin EM map from **C** (indicated by long arrows). **B.** Neighbor plot showing the spatial distribution of neighboring clathrin legs in the YxxΦ /[ED]xxxL[LI] coat (visualised as in **S3A** but integrating over a ~110 Å-thick slab). The white disc indicates the origin relative to which positions of neighbors were determined. Dotted lines outline clathrin polygons. Arrows indicate peaks where neighboring legs are frequently present marked according to whether they correspond to neighbors around a hexagon (H) or a pentagon (P). This neighbor plot was used to sort clathrin legs into hexagon and pentagon

bounding classes according to whether their neighbours were at H or P positions. **C.** EM maps of the hexagon- and pentagon-bounding classes combined from both YxxΦ and YxxΦ /[ED]xxxL[LI] coat datasets, coloured by resolution as in **Fig. S2A**. The maps are shown at different levels of sharpening to reveal secondary structure (B factor -500) or low-resolution features such as NTD domains and β2-appendages (B factor -100). The resolution drops sharply in the NTD-ankle region, demonstrating the mobility of these clathrin elements compared to the rest of the structure. **D.** The corresponding FSC plots.

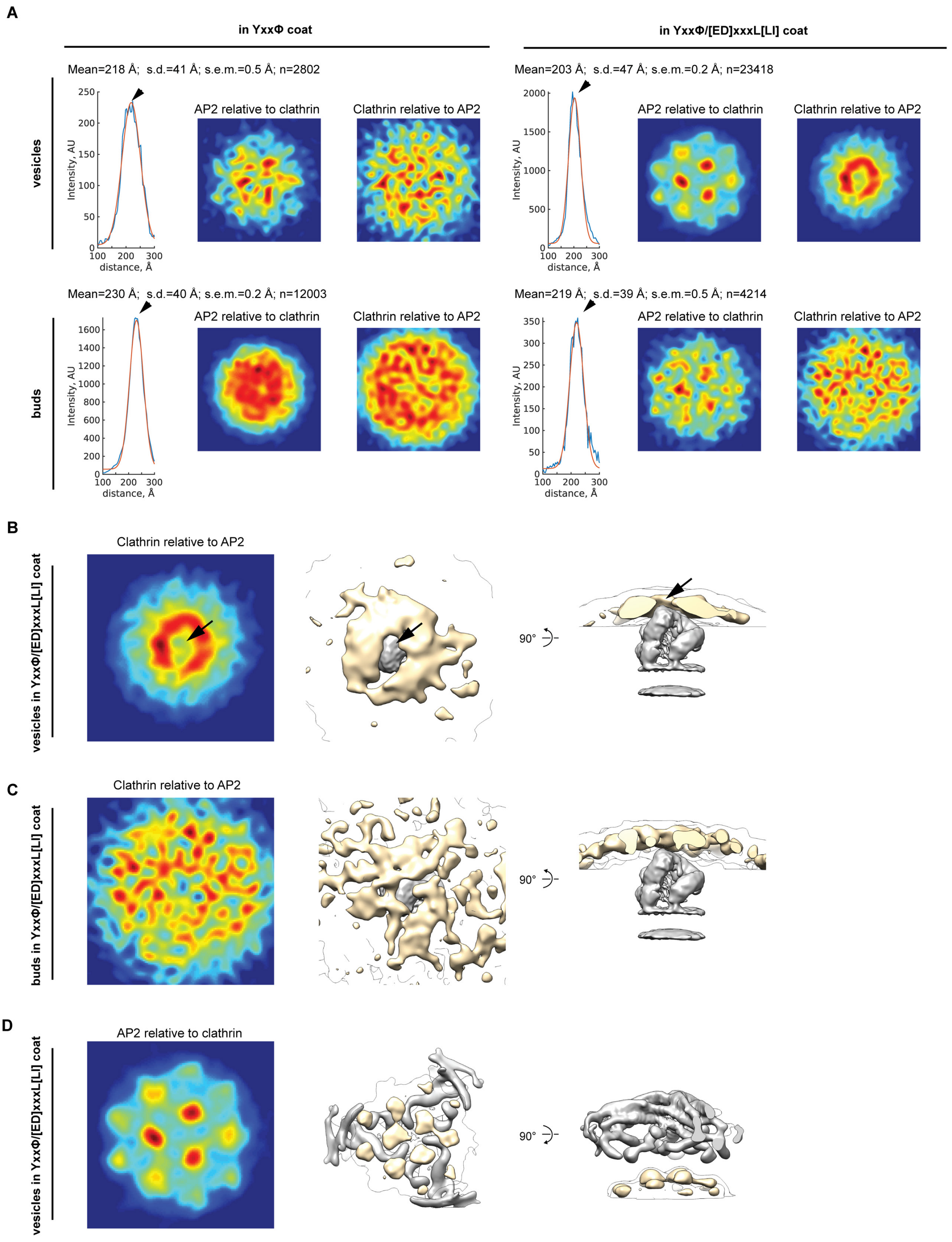

Fig. S5. Spatial relationship between AP2 and clathrin in coats. A. Neighbor plots showing the distribution of the center of AP2 relative to the clathrin tripod or the distribution of clathrin NTDs relative to the center of AP2 molecules. Plots are visualized as in **Fig S3A**, integrating 90 Å-thick slabs (for AP2 relative to clathrin) or 82 Å-thick slabs (for clathrin relative to AP2). Also shown are distributions of the radial distance between AP2 and clathrin calculated from the "AP2 relative to clathrin" neighbor plots (blue lines, see supplementary methods). Mean

and standard deviation (s.d.) were determined by fitting with a Gaussian distribution (red lines) and the standard error of the mean (s.e.m.) and the number of AP2 subtomograms considered (n) is marked. The distance between clathrin and AP2 is shorter in YxxΦ/[ED]xxxL[LI] cargo-containing coats than in YxxΦ cargo-containing coats, and is shorter in vesicles than in buds. AP2 begins to occupy preferred positions between clathrin NTDs at a distance between AP2 and clathrin shorter than ~220 Å, and this preference becomes pronounced at a distance of ~203 Å as seen in YxxΦ /[ED]xxxL[LI] coated vesicles. Corresponding changes are seen in the distribution of clathrin relative to AP2, with clathrin NTD being excluded from positions directly above AP2 molecules (see also panel **B**). **B-D.** Selected neighbor plots from **A** are additionally shown as isosurfaces that illustrate the most likely positions of neighboring molecules. The beige isosurface encloses 8% of relative subtomogram positions, the transparent outline encloses 40%. These isosurfaces are shown relative to the protein structures (grey surface). For example, the beige isosurface in B encloses the preferred location of the clathrin NTD relative to an AP2 molecule shown in grey. **B.** Clathrin NTDs are preferentially excluded from regions directly above AP2 in vesicles (indicated by arrows) where the distance between AP2 and clathrin is short enough for them to sterically exclude one another. **C.** Clathrin NTDs are randomly distributed in buds where the distance between AP2 and clathrin is large enough to avoid any steric exclusion. **D.** The preferred positions of AP2 coincide with the spaces between the clathrin NTDs – each space provides enough room to accommodate the tip of a single AP2.

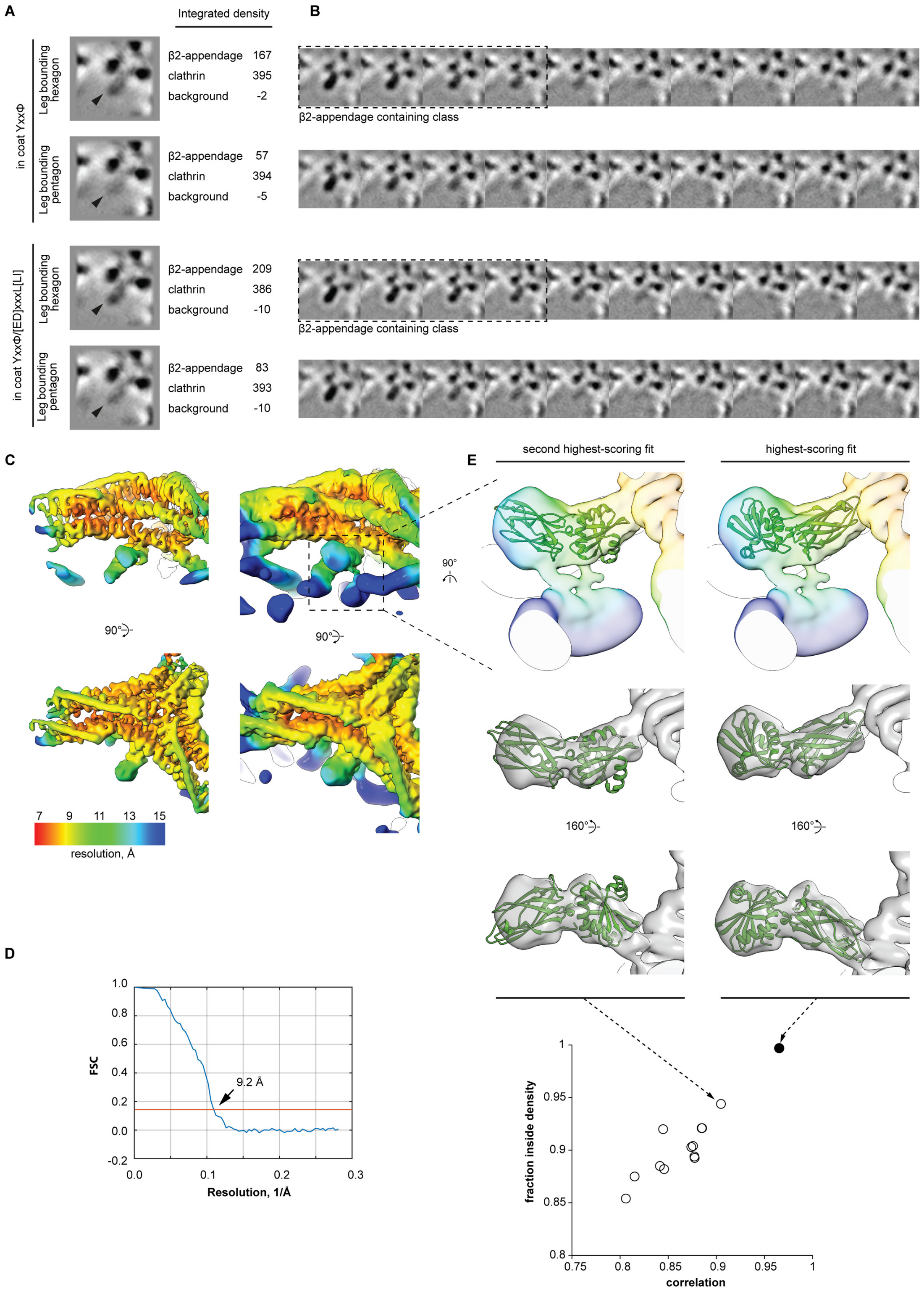

Fig. S6. Classification of β2-appendage enriched subtomograms and rigid body fitting of the atomic structure of β2-appendage. A. Slices through EM maps of clathrin legs bounding hexagons and pentagons. The β2-appendage density (arrowheads) is stronger in the hexagon-bounding leg than in the pentagon-bounding leg. The pixel density was integrated within an ellipsoidal mask placed on the β2-appendage, clathrin, or background, revealing higher

occupancy if the β2-appendage on legs bounding hexagons than bounding pentagons. We estimate that the occupancy is approximately 50% and 20% in legs bounding hexagons and pentagons, respectively. **B**. Subtomograms classified based on β2-appendage occupancy. Panels are arranged in rows by the polygon and coat types as in **A**. The datasets were split into ten equally-sized classes by the score of the principal component that represents the β2-appendage density. Classes of hexagon-bounding legs enriched by β2-appendage were selected using visual inspection (classes within dotted boxes), and the corresponding subtomograms were combined into a single dataset which was averaged to produce the EM map shown in **C**. **C**. EM map of clathrin leg bounding hexagon enriched in the β2-appendage. The map is shown at two different isosurface levels and is coloured by local resolution as in **Fig. S2A**. **D.** Corresponding FSC plot. **E.** Rigid body fit of the atomic model of the β2-appendage domain (PDB ID 1e42, [9]) into the EM map from **C** was assessed based on correlation and the fraction of the model within the density, revealing an outlying highest-scoring fit (filled circle, **lower panel**). The EM map and the fitted ribbon models for the highest-scoring and second highest-scoring fits are illustrated for two different orientations and isosurface levels. Only the highest scoring fit is consistent with the EM density (right column).

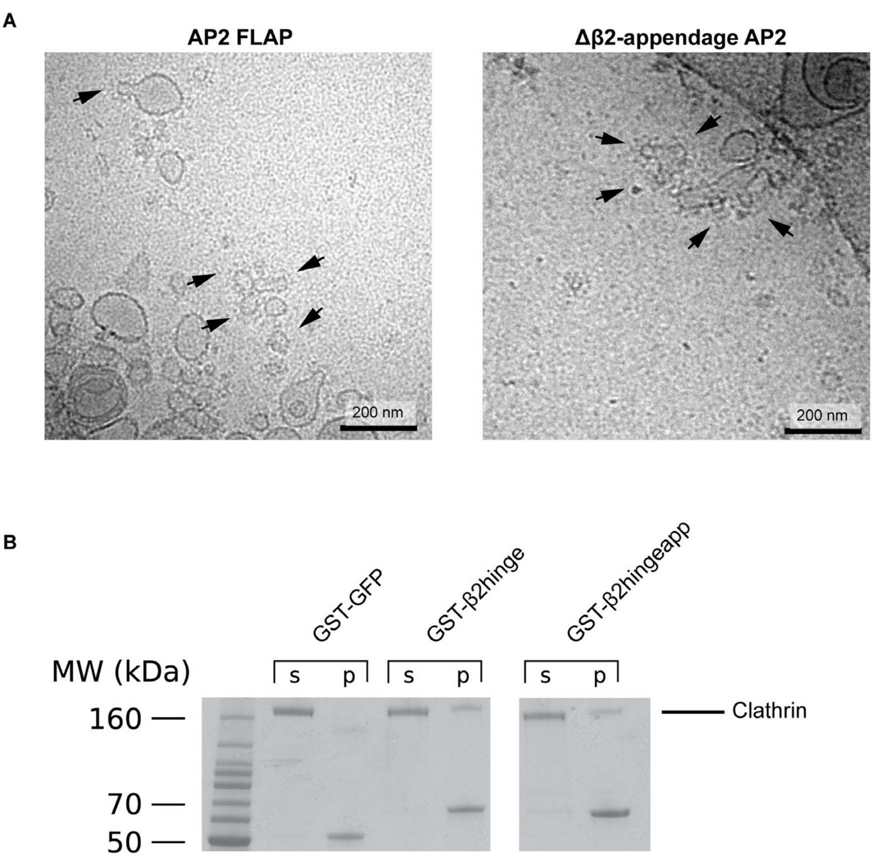

Fig. S7. Deletion of β2-appendage in AP2 FLAP does not preclude the formation of clathrin-coated buds. A. Cryo-EM screening images showing budding reactions on YxxΦ cargo-containing liposomes using AP2 FLAP and AP2 FLAP lacking the β2-appendage (Δβ2-appendage). Arrows indicate clathrin-coated buds formed in the budding reactions. **B.** Coomassie stained SDS-PAGE of glutathione sepharose pulldowns using GST-GFP (as a negative control for clathrin binding), GST-β2hinge or GST-β2hingeapp. Supernatant ('s') and pellet ('p'). GST-β2hinge and GST-β2hingeapp bind clathrin to a similar extent, suggesting that deletion of the β2-appendage does not affect clathrin recruitment in this system.

	AP2 in cytosol
EMDB/PDB ID	10747/6YAE
**Data collection and processing**	
Voltage (keV)	300
Microscope and Detector	Titan Krios, Falcon 3EC
Energy filter slit width (eV)	no
Electron exposure (e/ Å^2)	32.4
Defocus range (μm)	1.9-3.1
Movie recording	60 frames per exposure
Magnification	X75,000
Pixel size (Å)	1.065
Initial number of particles (no.)	119,744
Final number of particles (no.)	80,794
Symmetry	C1
Resolution at FSC threshold 0.143 (Å)	3.8
**Refinement**	
Initial model, PDB ID	2vgl
Model resolution (Å)	3.9
Map sharpening factor	-200
Non-hydrogen atoms	13707
Protein residues	1717
Ligands	0
R.m.s. deviations	
Bond length (Å)	0.005
Bond angle (°)	0.775
Validation	
Molprobity score	2.04
Clashscore	12.67
Poor rotamers (%)	0.07
Ramachandran plot	
Favoured (%)	93.60
Allowed (%)	6.28
Outliers (%)	0.12

Table S1. Cryo-electron microscopy data collection, refinement and validation statistics for single-particle structure of AP2 in solution.

	AP2<br>YxxΦ membrane	AP2/Clathrin<br>YxxΦ membrane	AP2/Clathrin<br>YxxΦ/[ED]xxxL[LI] membrane
Voltage (keV)		300	
Microscope and Detector		Titan Krios, Gatan Quantum K2	
Energy filter slit width (eV)		20	
Electron exposure (e/Å2) dose fractionation		~130, uniformly distributed over tilt series	
Defocus range (μm)	2.5-5.5	2.5-6.5	1.5-4.5
Tilt scheme (min/max, step)		-60°/+60, 3°, dose-symmetrical (Hagen scheme)	
Movie recording		10 frames per tilt	
Magnification (times)		X81,000	
Pixel size (Å)	1.780	1.780	1.787
Number of tomograms acquired/used (no.)	77/66	48/45	44/43
Traced membranes (no.) and lumenal diameter (mean±std)	1083 liposomes (99±46 nm) 244 tubules (19±7 nm)	397 buds (35±4 nm)<br>55 vesicles (70±10 nm)	96 buds (56±6 nm)<br>564 vesicles (56±10nm)

Table S2. Cryo-electron tomography data collection parameters for membrane-recruited AP2 and clathrin.

	AP2				Clathrin		
	on YxxΦ membrane		in YxxΦ coat	in YxxΦ/[ED]xxxL[LI] coat	hexagon-bounding	pentagon-bounding	hexagon-bounding, β2-appendage enriched
	monomer	dimer					
EMDB/PDB ID	10748/6YAF	10749	10750	10751/6YAH	10752	10753	10754/6YAI
Initial subtomograms (no.)	585,669		224,577	292,224		79,913[1]/118,958[2]	
Subtomograms after distance/cluster cleaning (no.)	112,237		25,694	29,139		20,917[1]/37,204[2]	
Subtomograms after cross-correlation threshold cleaning (no.)	100,556		14,805	27,630		18,371[1]/23,832[2]	
Subtomograms after symmetry expansion (no.)	n/a		n/a	n/a		55,131[1]/71,496[2]	
Final subtomogram (no.)	100,556	22,323	14,805	27,630	30,190	24,759	12,076
Symmetry	C1	C2	C1	C1	C1	C1	C1
Resolution at FSC threshold 0.143 (Å)	9.1	10.2	11.9	10.2	7.7	7.7	9.2
Resolution range (Å)	7-18	8.5-24	9-24	9-24	6-25	6-25	7.5-30

1. YxxΦ coat
2. YxxΦ/[ED]xxxL[LI] coat

Table S3. Subtomogram averaging image processing parameters and statistics for membrane-recruited AP2 and clathrin.
